# Scavenging Tumor Necrosis Factor α Does Not Affect Inhibition of Dentate Granule Cells Following In Vitro Entorhinal Cortex Lesion

**DOI:** 10.3390/cells10113232

**Published:** 2021-11-19

**Authors:** Dimitrios Kleidonas, Andreas Vlachos

**Affiliations:** 1 Department of Neuroanatomy, Institute of Anatomy and Cell Biology, Faculty of Medicine, University of Freiburg, 79104 Freiburg, Germany; dimitrios.kleidonas@anat.uni-freiburg.de; 2 Spemann Graduate School of Biology and Medicine (SGBM), University of Freiburg, 79104 Freiburg, Germany; 3 Faculty of Biology, University of Freiburg, 79104 Freiburg, Germany; 4 Center Brain Links Brain Tools, University of Freiburg, 79110 Freiburg, Germany; 5 Center for Basics in NeuroModulation (NeuroModulBasics), Faculty of Medicine, University of Freiburg, 79106 Freiburg, Germany

**Keywords:** entorhinal cortex lesion, denervation, TNFα, microglia, synaptic scaling, inhibition

## Abstract

Neurons that lose part of their afferent input remodel their synaptic connections. While cellular and molecular mechanisms of denervation-induced changes in excitatory neurotransmission have been identified, little is known about the signaling pathways that control inhibition in denervated networks. In this study, we used mouse entorhino-hippocampal tissue cultures of both sexes to study the role of the pro-inflammatory cytokine tumor necrosis factor α (TNFα) in denervation-induced plasticity of inhibitory neurotransmission. In line with our previous findings in vitro, an entorhinal cortex lesion triggered a compensatory increase in the excitatory synaptic strength of partially denervated dentate granule cells. Inhibitory synaptic strength was not changed 3 days after the lesion. These functional changes were accompanied by a recruitment of microglia in the denervated hippocampus, and experiments in tissue cultures prepared from TNF-reporter mice [*C57BL/6-Tg(TNFa-eGFP)*] showed increased TNFα expression in the denervated zone. However, inhibitory neurotransmission was not affected by scavenging TNFα with a soluble TNF receptor. In turn, a decrease in inhibition, i.e., decreased frequencies of miniature inhibitory postsynaptic currents, was observed in denervated dentate granule cells of microglia-depleted tissue cultures. We conclude from these results that activated microglia maintain the inhibition of denervated dentate granule cells and that TNFα is not required for the maintenance of inhibition after denervation.

## 1. Introduction

The balance of excitation and inhibition is essential for the proper function and for information processing in cortical circuits [1,2,3]. While feedforward and feedback circuits dynamically match recruited inhibition to afferent excitation and local network activity, synaptic plasticity maintains this balance over longer periods of time [4]. Homeostatic synaptic plasticity—a synaptic mechanism that is based on negative feedback mechanisms—is considered to play an important role in this context [5,6]. Work from the past two decades has identified several cellular and molecular mechanisms that mediate and modulate the ability of neurons to express the homeostatic plasticity of excitatory and inhibitory neurotransmission [7,8,9]. Nevertheless, the biological significance of homeostatic synaptic changes that occur under pathological conditions such as brain injury and neurodegeneration remains elusive.

Neurons that are not directly affected by a lesion but lose part of their afferent excitation following brain injury increase their excitatory synaptic strength in a compensatory manner [10,11,12,13,14,15]. Interestingly, the downscaling of inhibitory neurotransmission is not consistently observed in partially denervated networks [16,17,18,19]. In a recent study, we used the well-established model of in vitro entorhinal cortex lesion and found no changes in inhibitory neurotransmission onto dentate granule cells 3 days after the lesion [19]. GABAergic neurotransmission also remained unchanged under conditions in which glutamatergic neurotransmission was pharmacologically blocked (both in non-denervated and denervated neurons [19]). We therefore theorized that mechanisms exist that maintain the GABAergic synaptic set-point by preventing its homeostatic adjustment, i.e., denervation-induced downscaling of inhibition, even under conditions in which neurons cannot compensate via changes in excitatory neurotransmission.

The pro-inflammatory cytokine tumor necrosis factor alpha (TNFα) is an interesting candidate molecule in this context. TNFα mediates the homeostatic synaptic plasticity of excitatory synapses [20]. Notably, evidence has been provided that TNFα also affects inhibitory neurotransmission [21,22]. Since TNF signaling pathways have been linked to denervation-induced plasticity [15,23,24], we wondered whether TNFα coordinates denervation-induced changes in excitatory and inhibitory neurotransmission, i.e., synaptic excitation/inhibition balance in denervated networks. Our previous work demonstrated that TNFα maintains the increased excitatory synaptic strength of partially denervated dentate granule cells [23]. Here, we tested whether TNFα also maintains GABAergic neurotransmission, thus preventing the downscaling of inhibition. Furthermore, we tested for the role of microglia, the brain’s resident immune cells, which are a major source of TNFα in the central nervous system [25,26,27], by depleting microglia from tissue cultures and assessing inhibitory neurotransmission in intact and partially denervated dentate granule cells.

## 2. Materials and Methods

### 2.1. Ethics Statement

Mice were maintained in a 12 h light/dark cycle with food and water available ad libitum. Every effort was made to minimize the distress and pain of animals. Experimental procedures (X-17/07K; X-17/09C) were performed according to German animal welfare legislation and approved by the appropriate animal welfare committee and the animal welfare officer of the University of Freiburg.

### 2.2. Animals

Wild-type *C57BL/6J* and *C57BL/6-Tg(TNFa-eGFP)* [28] mice of both sexes were used in this study.

### 2.3. Preparation of Tissue Cultures

Organotypic entorhino-hippocampal tissue cultures were prepared at postnatal day 4–5 from mice of either sex as previously described [29]. The tissue cultures were transferred onto porous (0.4 µm pore size, hydrophilic PTFE) cell culture inserts with 30 mm diameter (Merck/Millipore, Darmstadt, Germany, Cat# PICM0RG50) for cultivation. The culturing medium consisted of 50% (*v*/*v*) minimum essential medium (MEM), 25% (*v*/*v*) basal medium eagle (BME), 25% (*v*/*v*) heat-inactivated normal horse serum (NHS), 2 mM GlutaMAX, 0.65% (*w*/*v*) glucose, 25 mM HEPES buffer solution, 0.1 mg/mL streptomycin, 100 U/mL penicillin and 0.15% (*w*/*v*) bicarbonate. The pH of the culturing medium was adjusted to 7.30 and tissue cultures were incubated for at least 18 days at 35 °C in a humidified atmosphere with 5% CO_2_. The culturing medium was replaced thrice a week at 2- and 3-day intervals. After the lesion, the medium was not replaced for 3 days until further experimental assessment.

### 2.4. Entorhinal Cortex Lesion

**Figure 1:**
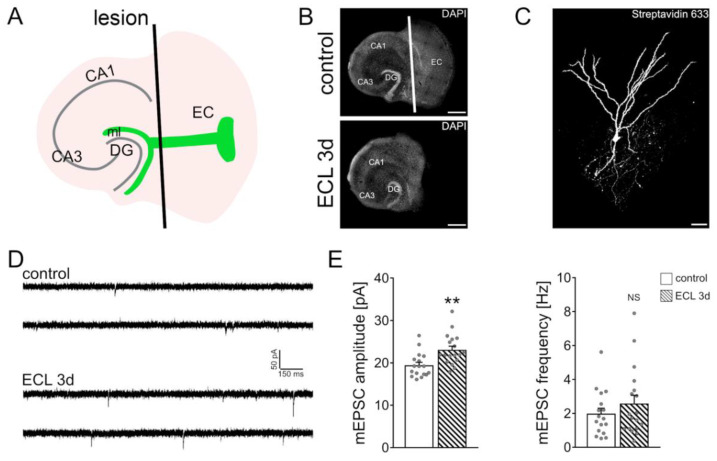
In vitro entorhinal cortex lesion induces a compensatory strengthening of excitatory synapses. (**A**) Schematic illustration of entorhinal cortex lesion (ECL) in vitro (DG, dentate gyrus; CA1, Cornu Ammonis Area 1; CA3, Cornu Ammonis Area 3; ml, molecular layer). (**B**) Mouse organotypic entorhino-hippocampal tissue cultures stained with DAPI (top, non-lesioned control tissue culture; bottom, denervated tissue culture). Scale bars 400 µm. (**C**) Recorded and post-hoc identified dentate granule cell (streptavidin 633; white). Scale bar 30 µm. (**D**,**E**) Sample traces and group data of AMPA receptor-mediated miniature excitatory postsynaptic currents (mEPSCs) recorded from dentate granule cells of non-denervated controls and age- and time-matched denervated tissue cultures 3 days after the lesion (control, *n* = 17 cells from 4 cultures; ECL 3d, *n* = 18 cells from 4 cultures; Mann–Whitney test; ** *p* < 0.01 NS, non-significant difference). For these graphs and for the rest of the graphs, values represent mean ± standard error of the mean (s.e.m.), and gray dots indicate individual data points.

In order to assure a complete and reproducible denervation of granule cells in the dentate gyrus, the entorhinal cortex was transected and removed from the culturing insert with a sterile scalpel blade (Figure 1A,B). All experiments were performed 3 days after the lesion.

### 2.5. Pharmacology

Organotypic entorhino-hippocampal tissue cultures (≥18 days in vitro) were treated with mouse recombinant soluble tumor necrosis factor receptor 1 (sTNFR1 10 μg/mL; R&D systems, Minneapolis, MN, USA, Cat# 425-R1-050), 20 min before the lesion and for 3 days. For the depletion of microglia in tissue cultures, PLX3397 (50 nM in DMSO; Axon MedChem, Groningen, Netherlands, Cat# 2501) was added to the culturing medium immediately after preparation and during the whole cultivation period. The respective control tissue cultures were treated with equal volume of dimethyl sulfoxide (DMSO).

### 2.6. Whole-Cell Patch-Clamp Recordings

Whole-cell patch-clamp recordings from dentate granule cells were carried out at 35 °C (1–6 cells per culture). The bath solution contained 126 mM NaCl, 2.5 mM KCl, 26 mM NaHCO_3_, 1.25 mM NaH_2_PO_4_, 2 mM CaCl_2_, 2 mM MgCl_2_, 10 mM glucose and was saturated with 95% O_2_/5% CO_2_. For AMPA receptor-mediated miniature excitatory postsynaptic currents (mEPSCs), the bath solution additionally contained 10 µM D-APV (Abcam, Cambridge, UK, Cat# ab120003), 10 µM bicuculline methiodide (BMI; Tocris, Bristol, UK, Cat# 2503) and 0.5 µM tetrodotoxin (TTX; Biotrend, Cologne, Germany, Cat# ARCD-0640-1). Miniature inhibitory postsynaptic current (mIPSC) recordings were conducted in bath solution additionally containing 10 µM D-APV, 10 µM CNQX (Abcam, Cambridge, UK, Cat# ab120017) and 0.5 µM TTX. Patch pipettes for AMPA receptor-mediated mEPSC recordings contained 126 mM K-gluconate, 10 mM HEPES, 4 mM KCl, 4 mM Mg-ATP, 0.3 mM GTP-Na_2_, 10 mM PO-Creatine and 0.1% (*w*/*v*) biocytin (Merck/Sigma-Aldrich, Darmstadt, Germany, Cat# B4261) (pH 7.25 with KOH, 290 mOsm with sucrose). For mIPSC recordings, patch pipettes contained 125 mM CsCl, 5 mM NaCl, 2 mM MgCl_2_, 2 mM Mg-ATP, 0.5 mM GTP-Na_2_, 0.1 mM EGTA and 10 mM HEPES (pH = 7.33 with CsOH, 274 mOsm with sucrose). Recordings were performed at a holding potential of −70 mV. Series resistance was monitored before and after each recording and recordings were discarded if the series resistance reached ≥30 MΩ.

### 2.7. Immunohistochemistry and Imaging

Tissue cultures were fixed in a solution containing 4% (*w*/*v*) paraformaldehyde (PFA) and 4% (*w*/*v*) sucrose in 0.01 M phosphate-buffered saline (PBS) for 1 h at room temperature, and then washed briefly with 0.01 PBS before storage in 0.01 M PBS at 4 °C. For immunohistochemistry, fixed tissue cultures were incubated for 1 h at room temperature in a blocking solution consisting of 10% (*v*/*v*) normal goat serum (NGS; Fisher Scientific, Schwerte, Germany, Cat# NC9270494) and 0.5% (*v*/*v*) Triton X-100 in 0.01 M PBS to reduce non-specific antibody binding. After blocking, the fixed tissue cultures were incubated for 48 h at 4 °C (while shaking) with a solution containing primary antibody against Iba1 (1:1000; Wako, Neuss, Germany, Cat# 019-19741) in 0.01 M PBS with 10% NGS and 0.1% Triton X-100. The fixed cultures were washed twice in 0.01 M PBS and incubated overnight at 4 °C with donkey anti-rabbit Alexa488-labeled secondary antibody (1:1000; Invitrogen, Waltham, MA, USA, Cat# A21206) in 0.01 M PBS with 10% NGS, 0.1% Triton X-100. DAPI (ThermoFisher, Waltham, MA, USA, Cat# 62248) nuclear staining was used to visualize cytoarchitecture (1:2000; in 0.01 M PBS for 15 min). Finally, the samples were washed thrice with 0.01 M PBS, transferred onto glass slides and mounted for visualization with anti-fading mounting medium (DAKO; Agilent, Santa Clara, CA, USA, Cat# S3023). Confocal images were acquired using a Leica TCS SP8 laser scanning microscope (Leica Microsystems, Wetzlar, Germany) with 20× (NA 0.75; Leica), 40× (NA 1.30; Leica) and 63× (NA 1.40; Leica) oil-submersion objectives.

### 2.8. Post-Hoc Identification of Recorded Neurons

For mEPSC recordings, biocytin-containing internal solution was used as described above. After recording, tissue cultures were fixed in a solution of 4% (*w*/*v*) paraformaldehyde (PFA) and 4% (*w*/*v*) sucrose in 0.01 M PBS for 1 h at room temperature. After washing with 0.01 M PBS, the fixed tissue was incubated for 1 h at room temperature in a blocking solution consisting of 10% (*v*/*v*) normal goat serum (NGS) and 0.5% (*v*/*v*) Triton X-100 in 0.01 M PBS. Biocytin-filled cells were stained with Alexa-633 conjugated Streptavidin (ThermoFisher Scientific, Waltham, MA, USA, Cat# S21375) in a dilution of 1:1000 in 0.01 M PBS with 10% NGS and 0.1% Triton X-100 overnight at 4 °C. DAPI staining was used to visualize cytoarchitecture (1:2000; in 0.01 M PBS for 15 min). Slices were then washed 3 times in 0.01 M PBS, transferred and mounted onto glass slides with anti-fading mounting medium (DAKO) for visualization.

### 2.9. Live-Cell Microscopy

Live-cell imaging of heterozygous *C57BL/6-Tg(TNFa-eGFP)* cultures, lesioned or non-lesioned, was performed with a Zeiss LSM800 (Zeiss, Jena, Germany) microscope equipped with a 10× water-immersion objective (NA 0.3; Carl Zeiss). Filter membranes with 3 cultures were placed in a 35 mm Petri dish containing pre-oxygenated imaging solution consisting of 50% (*v*/*v*) MEM, 25% (*v*/*v*) basal medium eagle, 50 mM HEPES buffer solution (25% *v*/*v*), 0.65% (*w*/*v*) glucose, 0.15% (*w*/*v*) bicarbonate, 0.1 mg/mL streptomycin, 100 U/mL penicillin, 2 mM GlutaMAX and 0.1 mM 6-Hydroxy-2,5,7,8-tetramethylchroman-2-carboxylic acid (trolox). The tissue cultures were kept at 35 °C during the imaging procedure. Laser intensity and detector gain were initially set to keep the fluorescent signal in a dynamic range throughout the experiment and imaging parameters were kept constant in all experiments. Confocal image stacks were stored as czi files.

### 2.10. Quantification and Statistics

Single cell recordings were analyzed using Clampfit 11 of the pClamp11 software package (Molecular Devices, San Jose, CA, USA). mEPSC and mIPSC properties were analyzed using the automated template search tool for event detection, which detects the events in an unbiased fashion. Confocal image stacks of heterozygous *C57BL/6-Tg(TNFa-eGFP)* cultures were processed and analyzed using the Fiji image processing package (available at http://imagej.net; accessed on 17 November 2021). From each stack, maximum intensity projection of 10 images was performed. The molecular layer of the dentate gyrus was manually defined as the region of interest (ROI) and the mean fluorescence intensity of each ROI was measured. For the assessment of microglia numbers, single plane images were analyzed. The molecular layer of the dentate gyrus was manually defined as the region of interest (ROI). After background subtraction (30 px), cell numbers were measured using the ‘Analyze Particles’ function of Fiji. Analysis was performed by the investigator blind to experimental conditions. Statistical comparisons were carried out using GraphPad Prism 7 (GraphPad software, San Diego, CA, USA). Non-parametric tests were performed (Mann–Whitney test and Kruskal–Wallis test followed by Dunn’s post hoc test) for the analysis of all experiments in the present study.

### 2.11. Digital Illustrations

Figures were prepared using the Affinity Designer (Serif Europe, Nottingham, UK) and the Adobe Photoshop (Adobe, San Jose, CA, USA) graphics software. Image brightness and contrast were adjusted. Figure 1A was created with BioRender (www.biorender.com; accessed on 17 November 2021).

## 3. Results

### 3.1. Partial Denervation of Dentate Granule Cells Induces a Compensatory Adjustment of Excitatory Synaptic Strength

The denervation-induced homeostatic synaptic plasticity of dentate granule cells was probed in mouse organotypic entorhino-hippocampal tissue cultures (≥18 days in vitro) as described before [11,12,19,23,24]. Slice cultures were transected from the rhinal fissure to the hippocampal fissure using a sterile scalpel blade (Figure 1A,B). To ensure complete and reproducible separation of the entorhinal cortex from the hippocampus, the entorhinal cortex was removed in every denervation experiment from the culturing insert. This procedure does not affect the target neurons in the dentate gyrus directly but leads to a reproducible partial denervation of distal dentate granule cell dendrites in the molecular layer of the dentate gyrus (Figure 1C). Three days after the lesion, individual dentate granule cells in the suprapyramidal blade of the dentate gyrus were patched and AMPA receptor-mediated miniature excitatory postsynaptic currents (mEPSCs) were recorded (Figure 1D). The amplitude of these events reflects excitatory synaptic strength. Consistent with our previous findings [11,12,19,23,24], a homeostatic increase in excitatory synaptic strength, i.e., increased mEPSC amplitudes, was observed 3 days after denervation (Figure 1E). These results confirm once more that in vitro denervation of dentate granule cells induces a robust compensatory strengthening of excitatory synapses.

### 3.2. TNFα Does Not Affect Inhibitory Neurotransmission onto Denervated Dentate Granule Cells

Our previous work showed that denervation-induced excitatory synaptic strengthening depends on TNFα [23,24]. However, the role of TNFα for inhibitory neurotransmission onto partially denervated dentate granule cells has not yet been addressed.

**Figure 2:**
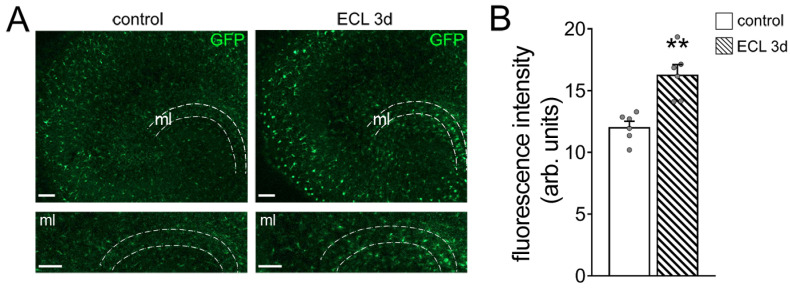
Entorhinal cortex lesion triggers *Tnfα* gene expression in the molecular layer of the dentate gyrus. (**A**) Representative images of tissue cultures prepared from TNF-reporter mice (*C57BL/6-Tg(TNFa-eGFP)*. The molecular layer (ml) is shown at higher magnification. Note increased GFP fluorescence 3 days after entorhinal cortex lesion (ECL). Scale bars 100 µm. (**B**) Group data of GFP fluorescence intensities in the ml of control and denervated tissue cultures (control, *n* = 6 cultures; ECL 3d, *n* = 6 cultures; Mann–Whitney test; ** *p* < 0.01).

Before assessing inhibitory neurotransmission, we confirmed that entorhinal cortex lesion triggers *Tnfα* gene expression in the denervated zone [23]. In these experiments, we used tissue cultures prepared from a TNF-reporter mouse line, which expresses enhanced green fluorescent protein (GFP) under the control of the TNF promoter [28]. Indeed, a significant increase in the GFP signal was observed in the molecular layer of denervated tissue cultures, thus confirming increased *Tnfα* gene expression in the denervated zone following in vitro entorhinal cortex lesion (Figure 2A,B).

**Figure 3:**
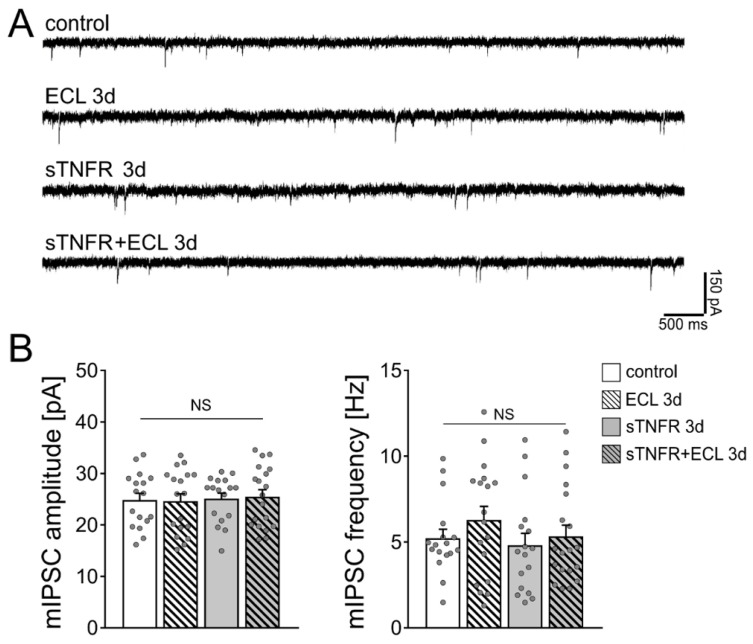
Scavenging TNFα with a soluble TNF receptor does not affect the strength of inhibitory synapses on dentate granule cells. (**A**) Sample traces of GABA receptor-mediated miniature inhibitory postsynaptic currents (mIPSCs) recorded from dentate granule cells of vehicle-only treated controls, denervated tissue cultures and tissue cultures treated with soluble TNF receptor (sTNFR, 10 µg/mL). (**B**) Group data of GABA receptor-mediated mIPSC recordings (untreated control, *n* = 17 cells from 5 cultures; untreated ECL 3d, *n* = 18 cells from 4 cultures; sTNFR 3d, *n* = 17 cells from 4 cultures; sTNFR + ECL 3d, *n* = 19 cells from 4 cultures; Kruskal–Wallis test followed by Dunn’s post hoc test; NS, non-significant difference).

Next, GABA receptor-mediated miniature inhibitory postsynaptic currents (mIPSCs) were recorded from individual control and denervated dentate granule cells of another set of wild-type cultures (Figure 3A). Three days after denervation, no significant differences in inhibitory synaptic strength were observed between the two groups (Figure 3B), thus corroborating our earlier findings on unaltered inhibitory neurotransmission following in vitro denervation [19].

We then used soluble TNFα receptor (sTNFR; 10 µg/mL) to scavenge TNFα. Our previous work showed that denervated dentate granule cells do not show increased excitatory synaptic strength 3 days after denervation in the presence of sTNFR [23]. sTNFR was added shortly before the entorhinal cortex lesion to the culturing medium for 3 days. Figure 3B shows that mIPSC amplitudes and frequencies were comparable in control and lesioned sTNFR-treated tissue cultures 3 days after the lesion. We conclude from these results that scavenging TNFα both in unlesioned control cultures and following denervation does not affect mIPSC properties of dentate granule cells.

### 3.3. Microglia Maintain Inhibition of Partially Denervated Dentate Granule Cells

**Figure 4:**
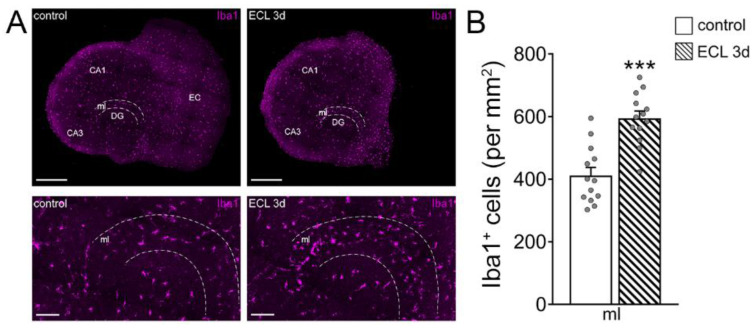
Recruitment of microglia in the denervated region. (**A**) Representative images of non-denervated controls and age- and time-matched denervated tissue cultures 3 days after entorhinal cortex lesion (ECL), stained for the microglial marker Iba1. The molecular layer (ml) is shown at higher magnification. Scale bars (top) 400 µm (bottom) 100 µm (DG, dentate gyrus; CA1, Cornu Ammonis Area 1; CA3, Cornu Ammonis Area 3). (**B**) Group data of the number of Iba1^+^ cells in the ml of DG in intact and denervated tissue cultures (control, *n* = 13 cultures; ECL 3d, *n* = 13 cultures; Mann–Whitney test; *** *p* < 0.001).

To confirm and extend these findings, we tested for the role of microglia on inhibitory neurotransmission in our experimental setting, also considering that microglia are a major source of TNFα in the central nervous system [25]. Immunostainings for the microglial marker Iba1 revealed a higher number of microglial cells in the denervated zone, suggesting an involvement of microglia in denervation-induced plasticity (Figure 4A,B; [30]).

**Figure 5:**
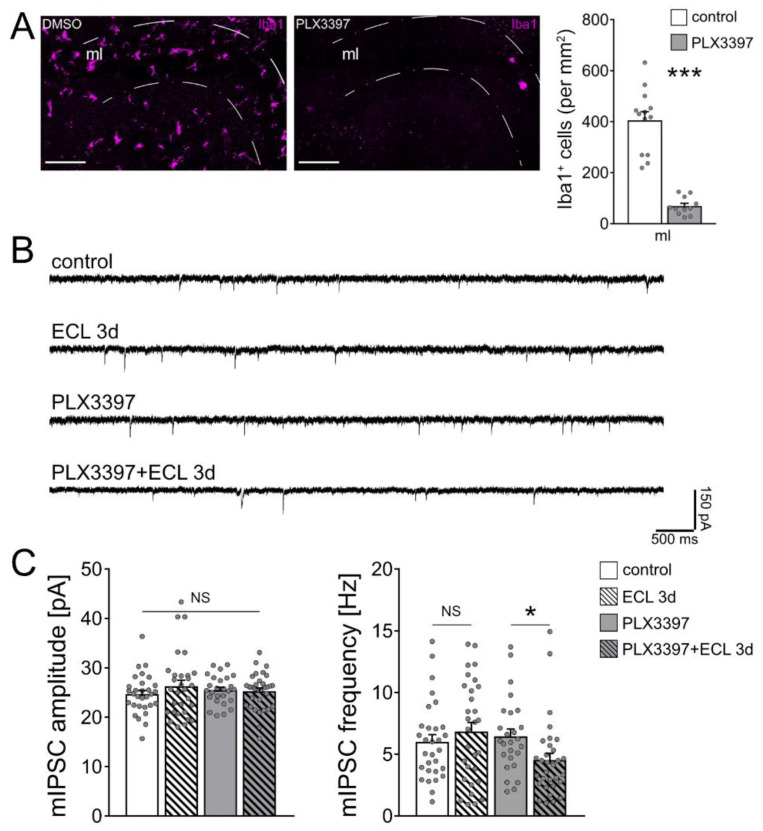
Microglia maintain inhibition onto partially denervated dentate granule cells. (**A**) Representative images of vehicle-only (i.e., dimethyl sulfoxide, DMSO) treated controls and PLX3397 (50 nM) treated tissue cultures, stained for the microglial marker Iba1, and group data of numbers of Iba1^+^ cells in the molecular layer (ml) of the dentate gyrus (control, *n* = 13 cultures; PLX3397, *n* = 11 cultures; Mann–Whitney test; *** *p* < 0.001). Scale bar 100 µm. (**B**) Sample traces of GABA receptor-mediated miniature inhibitory postsynaptic currents (mIPSCs) recorded from dentate granule cells in the respective groups. (**C**) Group data of GABA receptor-mediated mIPSC recordings (untreated control, *n* = 30 cells from 8 cultures; untreated ECL 3d, *n* = 30 cells from 6 cultures; PLX3397, *n* = 26 cells from 6 cultures; PLX3397 + ECL 3d, *n* = 32 cells from 7 cultures; Kruskal–Wallis test followed by Dunn’s post hoc test; * *p* < 0.05; NS, non-significant difference).

To test for the role of microglia on inhibitory neurotransmission, microglia were depleted from tissue cultures using the colony stimulating factor 1 receptor (CSF1R) antagonist PLX3397 [31,32,33]. Tissue cultures were exposed to 50 nM PLX3397 right after preparation and kept in the same concentration of PLX3397 until and during experimental assessment. Using this approach, the vast majority of microglia were successfully depleted from tissue cultures (Figure 5A). We previously showed that the depletion of microglia from tissue cultures does not affect cell viability and basic functional and structural properties of neurons [33]. Consistent with this suggestion, no changes in the mean series resistance and capacitance of dentate granule cells were observed between the groups (R_control_ = 7.304 MΩ ± 0.45: R_PLX3397_ = 7.959 MΩ ± 0.46; C_control_ = 20.000 pF ± 1.89: C_PLX3397_ = 17.530 pF ± 1.49). Once more, mIPSCs were recorded from individual dentate granule cells and we found no significant differences in the amplitudes and frequencies between non-depleted control and denervated tissue cultures (Figure 5B,C). While the depletion of microglia had no apparent effects on baseline mIPSC properties, a significant reduction in mIPSC frequencies was observed 3 days after denervation in the microglia-depleted tissue cultures (Figure 5B,C). We conclude from these results that microglia maintain inhibitory synaptic strength of dentate granule cells following in vitro partial denervation.

## 4. Discussion

The results of this study show that scavenging TNFα for 3 days with sTNFR does not affect inhibitory synapses, i.e., mIPSC properties, in cultured dentate granule cells, both under control conditions and following partial denervation. However, a denervation-induced reduction in inhibition, i.e., decreased mean mIPSC frequency, is observed when microglia are depleted from tissue cultures. This finding is consistent with a compensatory adjustment of inhibition following partial denervation, i.e., decreased inhibition in response to the loss of afferent excitation. Since this homeostatic adjustment of inhibition did not occur in the presence of microglia or when TNFα was scavenged, we conclude that microglia maintain the inhibition of partially denervated dentate granule cells in a TNFα-independent manner.

The ability of neurons to compensate for perturbations in afferent excitation is considered fundamental for normal brain function [5,6]. During the past few decades, several molecular mechanisms, which mediate homeostatic synaptic plasticity, have been identified [7,8,9]. It is now well-established that activity deprivation triggers the synaptic upscaling of excitatory neurotransmission and downscaling of synaptic inhibition [34,35,36,37]. However, the biological significance of compensatory changes at excitatory and inhibitory synapses remains unclear. For example, it is currently unknown whether homeostatic synaptic mechanisms support or obstruct the network integration of newly born dentate granule cells [38] and the recovery of synaptic connectivity after brain injury [39]. Likewise, the mechanisms that coordinate homeostatic changes at excitatory and inhibitory synapses at the single cell level are subject to further investigation.

Evidence has been provided that distinct mechanisms regulate the homeostasis of excitatory and inhibitory synapses. For example, CaMKIV-/pCREB-mediated signaling pathways mediate compensatory changes of excitatory synaptic strength (and intrinsic cellular properties) but not inhibitory neurotransmission [40]. Similarly, Clptm1 affects GABA_A_ receptors without changing AMPAR-mediated currents [41]. Conversely, the proinflammatory cytokine TNFα has been linked to the ability of neurons to express both excitatory and inhibitory synaptic plasticity [21,22,42], thus indicating that TNFα could be a key mediator of coordinated excitatory and inhibitory homeostatic synaptic changes.

Indeed, our previous work showed that TNFα maintains increased excitatory synaptic strength following an in vitro entorhinal cortex lesion [23,24]. Specifically, the initial denervation-induced compensatory increase in excitatory synaptic strength returned to baseline 3 days post lesion when TNFα was scavenged with 10 µg/mL sTNFR [23]. These results suggest that TNFα is required for the maintenance of denervation-induced synaptic strengthening. In a follow-up study, we assessed denervation-induced homeostatic changes of inhibitory neurotransmission and found no significant changes after the lesion [19]. Accordingly, we theorized that TNFα could be one of the major homeostatic factors that maintain increased excitatory and unchanged inhibitory synaptic neurotransmission in denervated neural networks. While we were able to reproduce our earlier findings on denervation-induced synaptic changes, the results of the present study showed that scavenging TNFα with soluble TNFR (sTNFR) does not trigger the downscaling of inhibitory synapses after denervation. Although we cannot exclude that TNFα-dependent changes in inhibitory neurotransmission may occur at a different time point after denervation, the results of the present study demonstrate that TNFα per se is not a key mediator of synchronous homeostatic changes at excitatory and inhibitory synapses, at least not in the denervated dentate gyrus 3 days after in vitro entorhinal cortex lesion.

Possible concentration-dependent effects of TNFα must be considered in this context [43,44,45]. For instance, low concentrations of TNFα reduce inhibitory neurotransmission in dissociated cultures [21,22], while high concentrations of TNFα—as seen after in vivo administration of bacterial lipopolysaccharide (LPS)—seem to enhance inhibitory neurotransmission [46]. While it is not trivial to measure the exact concentration of endogenous TNFα at synaptic sites, denervation seems to be a mild TNFα-inducing stimulus, specifically when compared to the exposure of tissue cultures to LPS [28]. It is therefore interesting to speculate that very low concentrations of TNFα may affect excitatory but not inhibitory neurotransmission. At a higher concentration, TNFα reduces inhibition [21,22], while very high concentrations of TNFα might increase synaptic inhibition [46]. This suggestion is supported by our previous work on the dose-dependent effects of TNFα in excitatory synaptic plasticity [43], where low concentrations of TNFα promoted LTP—possibly by reducing inhibition—and high concentrations blocked the ability of neurons to express LTP—possibly by increasing inhibition. Apparently, more work is required to clarify the concentration-dependent effects of TNFα on inhibitory neurotransmission. It will also be important to test for input-/synapse-specific effects of TNFα on dendritic vs. somatic inhibition in this context, since in the present study increased TNFα expression was observed mainly in the denervated molecular layer, where the distal dendrites of denervated granule cells are located [11,23,39].

Considering that distinct mechanisms seem to regulate excitatory and inhibitory neurotransmission, we tested for the role of microglia in our experimental setting. In line with previous in vivo work [30], increased numbers of microglia were observed in the molecular layer of denervated tissue cultures. Interestingly, when the majority of microglia was depleted from tissue cultures using an established protocol, i.e., PLX3397 [31,32,33], a homeostatic decrease in inhibition, i.e., a reduction in mIPSC frequencies, was demonstrated in partially denervated dentate granule cells. Notably, no differences in mIPSC properties were observed between non-depleted and microglia-depleted tissue cultures under baseline conditions, indicating that the presence of microglia is not required for dentate granule cells to obtain and maintain their inhibitory synaptic set-point under control conditions. However, the denervation-induced activation of microglia seems mandatory for the maintenance of inhibition following denervation. These findings suggest that microglia prevent the synaptic downscaling of inhibition in partially denervated neural networks. It is unlikely that TNFα mediates this function of microglia, since sTNFR (10 µg/mL), which scavenges TNFα (regardless of its source), did not affect inhibitory neurotransmission under control conditions and 3 days following in vitro denervation.

Nevertheless, it should be noted in this context that astrocytic and neuronal sources of TNFα have been reported [47,48,49]. Our experiments using TNF-reporter mice did not provide any evidence for a neuronal source of *Tnfα*, since no changes in GFP signal were observed in the granule cell layer. However, at this point we cannot fully exclude astrocytic sources of TNFα that may have escaped our detection in the denervated molecular layer, also considering our previous work that indicated changes in astrocytic TNFα following an entorhinal cortex lesion in vitro [23]. We are confident that future work in organotypic tissue cultures will support the identification of the cellular and molecular mechanisms through which microglia and astrocytes coordinate homeostatic changes in synaptic excitation/inhibition balance under physiological conditions and following partial denervation.

## Data Availability

All data obtained during the present study are available from the corresponding author upon reasonable request.

## References

[1] Dehghani N., Peyrache A., Telenczuk B., Le Van Quyen M., Halgren E., Cash S.S., Hatsopoulos N.G., Destexhe A. (2016). Dynamic Balance of Excitation and Inhibition in Human and Monkey Neocortex. Sci. Rep..

[2] Rubin R., Abbott L.F., Sompolinsky H. (2017). Balanced excitation and inhibition are required for high-capacity, noise-robust neuronal selectivity. Proc. Natl. Acad. Sci. USA.

[3] Zhou S., Yu Y. (2018). Synaptic E-I Balance Underlies Efficient Neural Coding. Front. Neurosci..

[4] Sprekeler H. (2017). Functional consequences of inhibitory plasticity: Homeostasis, the excitation-inhibition balance and beyond. Curr. Opin. Neurobiol..

[5] Turrigiano G. (2012). Homeostatic synaptic plasticity: Local and global mechanisms for stabilizing neuronal function. Cold Spring Harb. Perspect. Biol..

[6] Keck T., Hübener M., Bonhoeffer T. (2017). Interactions between synaptic homeostatic mechanisms: An attempt to reconcile BCM theory, synaptic scaling, and changing excitation/inhibition balance. Curr. Opin. Neurobiol..

[7] Pozo K., Goda Y. (2010). Unraveling mechanisms of homeostatic synaptic plasticity. Neuron.

[8] Fernandes D., Carvalho A.L. (2016). Carvalho. Mechanisms of homeostatic plasticity in the excitatory synapse. J. Neurochem..

[9] Keck T., Toyoizumi T., Chen L., Doiron B., Feldman D.E., Fox K., Gerstner W., Haydon P.G., Hübener M., Lee H.-K. (2017). Integrating Hebbian and homeostatic plasticity: The current state of the field and future research directions. Philos. Trans. R. Soc. B Biol. Sci..

[10] Yu X., Chung S., Chen D.-Y., Wang S., Dodd S.J., Walters J.R., Isaac J.T., Koretsky A.P. (2012). Thalamocortical inputs show post-critical-period plasticity. Neuron.

[11] Vlachos A., Becker D., Jedlicka P., Winkels R., Roeper J., Deller T. (2012). Entorhinal denervation induces homeostatic synaptic scaling of excitatory postsynapses of dentate granule cells in mouse organotypic slice cultures. PLoS ONE.

[12] Vlachos A., Ikenberg B., Lenz M., Becker D., Reifenberg K., Orth C.B., Deller T. (2013). Synaptopodin regulates denervation-induced homeostatic synaptic plasticity. Proc. Natl. Acad. Sci. USA.

[13] Becker D., Ikenberg B., Schiener S., Maggio N., Vlachos A. (2014). NMDA-receptor inhibition restores Protease-Activated Receptor 1 (PAR1) mediated alterations in homeostatic synaptic plasticity of denervated mouse dentate granule cells. Neuropharmacology.

[14] Keck T., Keller G.B., Jacobsen R.I., Eysel U., Bonhoeffer T., Hübener M. (2013). Synaptic scaling and homeostatic plasticity in the mouse visual cortex in vivo. Neuron.

[15] Barnes S., Franzoni E., Jacobsen R.I., Erdelyi F., Szabo G., Clopath C., Keller G.B., Keck T. (2017). Deprivation-Induced Homeostatic Spine Scaling In Vivo Is Localized to Dendritic Branches that Have Undergone Recent Spine Loss. Neuron.

[16] Deller T., Frotscher M., Nitsch R. (1995). Morphological evidence for the sprouting of inhibitory commissural fibers in response to the lesion of the excitatory entorhinal input to the rat dentate gyrus. J. Neurosci..

[17] Simburger E., Plaschke M., Kirsch J., Nitsch R. (2000). Distribution of the receptor-anchoring protein gephyrin in the rat dentate gyrus and changes following entorhinal cortex lesion. Cereb. Cortex.

[18] Dinocourt C., Aungst S., Yang K., Thompson S.M. (2011). Homeostatic increase in excitability in area CA1 after Schaffer collateral transection in vivo. Epilepsia.

[19] Lenz M., Galanis C., Kleidonas D., Fellenz M., Deller T., Vlachos A. (2019). Denervated mouse dentate granule cells adjust their excitatory but not inhibitory synapses following in vitro entorhinal cortex lesion. Exp. Neurol..

[20] Stellwagen D., Malenka R.C. (2006). Synaptic scaling mediated by glial TNF-alpha. Nature.

[21] Stellwagen D., Beattie E.C., Seo J.Y., Malenka R.C. (2005). Differential regulation of AMPA receptor and GABA receptor trafficking by tumor necrosis factor-alpha. J. Neurosci..

[22] Pribiag H., Stellwagen D. (2013). TNF-alpha downregulates inhibitory neurotransmission through protein phosphatase 1-dependent trafficking of GABA(A) receptors. J. Neurosci..

[23] Becker D., Zahn N., Deller T., Vlachos A. (2013). Tumor necrosis factor alpha maintains denervation-induced homeostatic synaptic plasticity of mouse dentate granule cells. Front. Cell. Neurosci..

[24] Becker D., Deller T., Vlachos A. (2015). Tumor necrosis factor (TNF)-receptor 1 and 2 mediate homeostatic synaptic plasticity of denervated mouse dentate granule cells. Sci. Rep..

[25] Goldmann T., Wieghofer P., Müller P.-F., Wolf Y., Varol D., Yona S., Brendecke S.M., Kierdorf K., Staszewski O., Datta M. (2013). A new type of microglia gene targeting shows TAK1 to be pivotal in CNS autoimmune inflammation. Nat. Neurosci..

[26] Welser-Alves J.V., Milner R. (2013). Microglia are the major source of TNF-alpha and TGF-beta1 in postnatal glial cultures; regulation by cytokines, lipopolysaccharide, and vitronectin. Neurochem. Int..

[27] Prinz M., Masuda T., Wheeler M.A., Quintana F.J. (2021). Microglia and Central Nervous System-Associated Macrophages-From Origin to Disease Modulation. Annu. Rev. Immunol..

[28] Lenz M., Eichler A., Kruse P., Strehl A., Rodriguez-Rozada S., Goren I., Yogev N., Frank S., Waisman A., Deller T. (2020). Interleukin 10 Restores Lipopolysaccharide-Induced Alterations in Synaptic Plasticity Probed by Repetitive Magnetic Stimulation. Front. Immunol..

[29] Del Turco D., Deller T. (2007). Organotypic entorhino-hippocampal slice cultures—A tool to study the molecular and cellular regulation of axonal regeneration and collateral sprouting in vitro. Methods Mol. Biol..

[30] Rappert A., Bechmann I., Pivneva T., Mahlo J., Biber K., Nolte C., Kovac A.D., Gerard C., Boddeke H.W.G.M., Nitsch R. (2004). CXCR3-dependent microglial recruitment is essential for dendrite loss after brain lesion. J. Neurosci..

[31] Han J., Harris R.A., Zhang X.-M. (2017). An updated assessment of microglia depletion: Current concepts and future directions. Mol. Brain.

[32] Coleman L.G., Zou J., Crews F.T. (2020). Microglial depletion and repopulation in brain slice culture normalizes sensitized proinflammatory signaling. J. Neuroinflam..

[33] Eichler A., Kleidonas D., Turi Z., Kirsch M., Pfeifer D., Masuda T., Prinz M., Lenz M., Vlachos A. (2021). Microglia mediate synaptic plasticity induced by 10 Hz repetitive magnetic stimulation. bioRxiv.

[34] Turrigiano G.G., Leslie K.R., Desai N.S., Rutherford L.C., Nelson S. (1998). Activity-dependent scaling of quantal amplitude in neocortical neurons. Nature.

[35] Thiagarajan T.C., Lindskog M., Tsien R.W. (2005). Adaptation to synaptic inactivity in hippocampal neurons. Neuron.

[36] Swanwick C.C., Murthy N.R., Kapur J. (2006). Activity-dependent scaling of GABAergic synapse strength is regulated by brain-derived neurotrophic factor. Mol. Cell. Neurosci..

[37] Kilman V., van Rossum M.C., Turrigiano G.G. (2002). Activity deprivation reduces miniature IPSC amplitude by decreasing the number of postsynaptic GABA(A) receptors clustered at neocortical synapses. J. Neurosci..

[38] Strehl A., Galanis C., Radic T., Schwarzacher S.W., Deller T., Vlachos A. (2018). Dopamine Modulates Homeostatic Excitatory Synaptic Plasticity of Immature Dentate Granule Cells in Entorhino-Hippocampal Slice Cultures. Front. Mol. Neurosci..

[39] Vlachos A., Helias M., Becker D., Diesmann M., Deller T. (2013). NMDA-receptor inhibition increases spine stability of denervated mouse dentate granule cells and accelerates spine density recovery following entorhinal denervation in vitro. Neurobiol. Dis..

[40] Joseph A., Turrigiano G.G. (2017). All for One But Not One for All: Excitatory Synaptic Scaling and Intrinsic Excitability Are Coregulated by CaMKIV, Whereas Inhibitory Synaptic Scaling Is Under Independent Control. J. Neurosci..

[41] Ge Y., Kang Y., Cassidy R., Moon K.-M., Lewis R., Wong R.O., Foster L.J., Craig A.M. (2018). Clptm1 Limits Forward Trafficking of GABAA Receptors to Scale Inhibitory Synaptic Strength. Neuron.

[42] Beattie E.C., Stellwagen D., Morishita W., Bresnahan J.C., Ha B.K., Von Zastrow M., Beattie M.S., Malenka R.C. (2002). Malenka. Control of synaptic strength by glial TNFalpha. Science.

[43] Maggio N., Vlachos A. (2018). Tumor necrosis factor (TNF) modulates synaptic plasticity in a concentration-dependent manner through intracellular calcium stores. J. Mol. Med..

[44] Bourgognon J.M., Cavanagh J. (2020). The role of cytokines in modulating learning and memory and brain plasticity. Brain Neurosci. Adv..

[45] Santello M., Volterra A. (2012). TNFalpha in synaptic function: Switching gears. Trends Neurosci..

[46] Tang B., Jiang J., Wang L., Misrani A., Huo Q., Han Y., Long C., Yang L. (2020). Microglial activation results in neuron-type-specific increase in mPFC GABAergic transmission and abnormal behavior in mice. bioRxiv.

[47] Lau L.T., Yu A.C. (2001). Astrocytes produce and release interleukin-1, interleukin-6, tumor necrosis factor alpha and interferon-gamma following traumatic and metabolic injury. J. Neurotrauma.

[48] Rodgers K., Lin Y., Langan T.J., Iwakura Y., Chou R.C. (2020). Innate Immune Functions of Astrocytes are Dependent Upon Tumor Necrosis Factor-Alpha. Sci. Rep..

[49] Park K.M., Bowers W.J. (2010). Tumor necrosis factor-alpha mediated signaling in neuronal homeostasis and dysfunction. Cell. Signal..

